# Supplementary material for: Structural basis of MICAL autoinhibition
Source: Nat Commun. 2024 Nov 12;15:9810. doi: 10.1038/s41467-024-54131-2 (PMC11557892; doi:10.1038/s41467-024-54131-2)
Supplement: Supplementary file 1 — Supplementary information [file 41467_2024_54131_MOESM1_ESM.pdf]

Supplementary Information

Structural basis of MICAL autoinhibition and activation

Matej Horvath[1,2], Adam Schrofel[1,2], Karolina Kowalska[1,2], Jan Sabo[3], Jonas Vlasak[1,2], Farahdokht Nourisanami[1,2], Margarita Sobol[1,2], Daniel Pinkas[4], Krystof Knapp[1,2], Nicola Koupilova[1,2], Jiri Novacek[4], Vaclav Veverka[1,5], Zdenek Lansky[3] and Daniel Rozbesky[1,2*]

Supplementary Figure 1

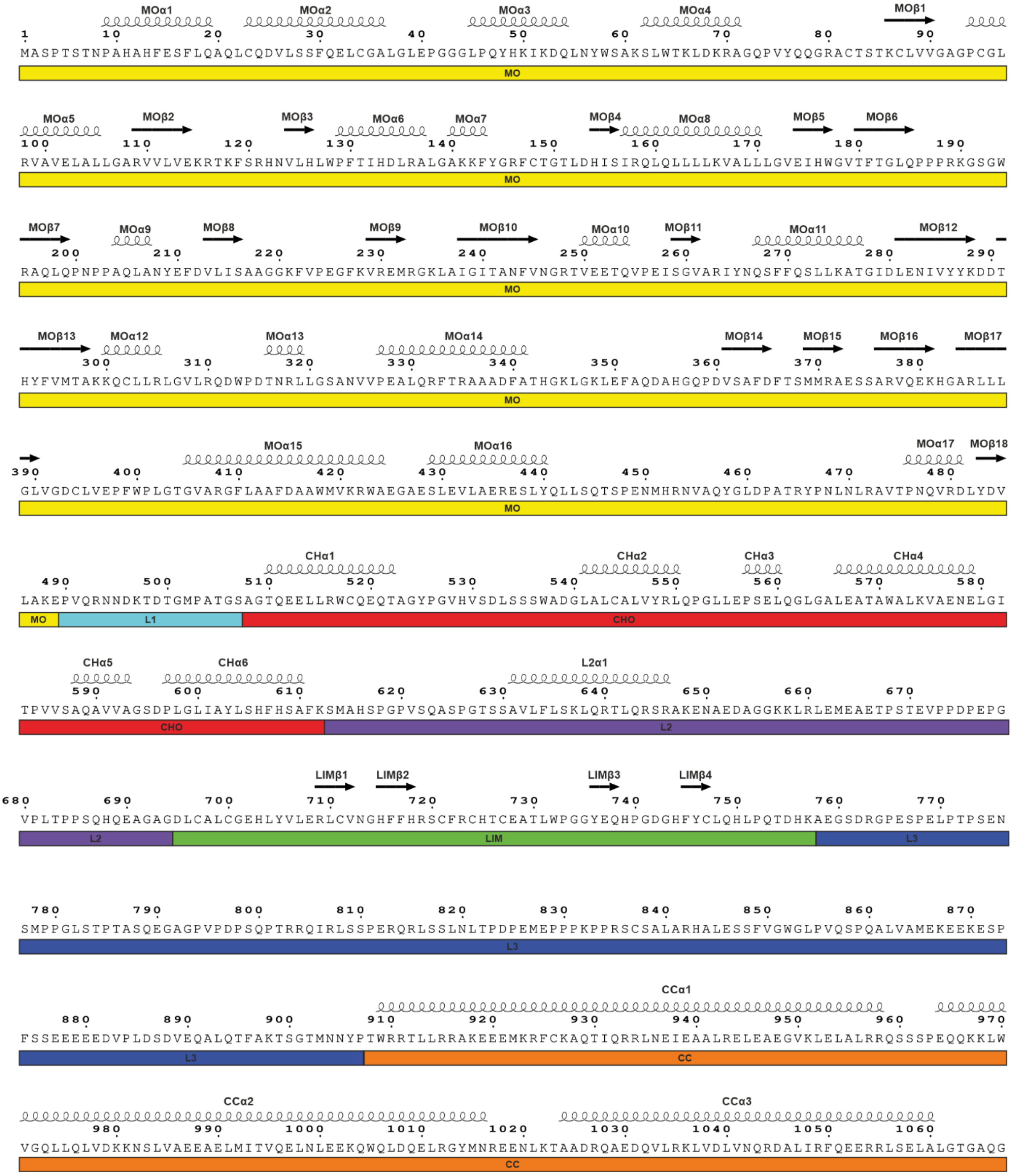

Supplementary Figure 1. Secondary structure elements and sequence of human MICAL1 generated by ESPript[53].

Supplementary Figure 2

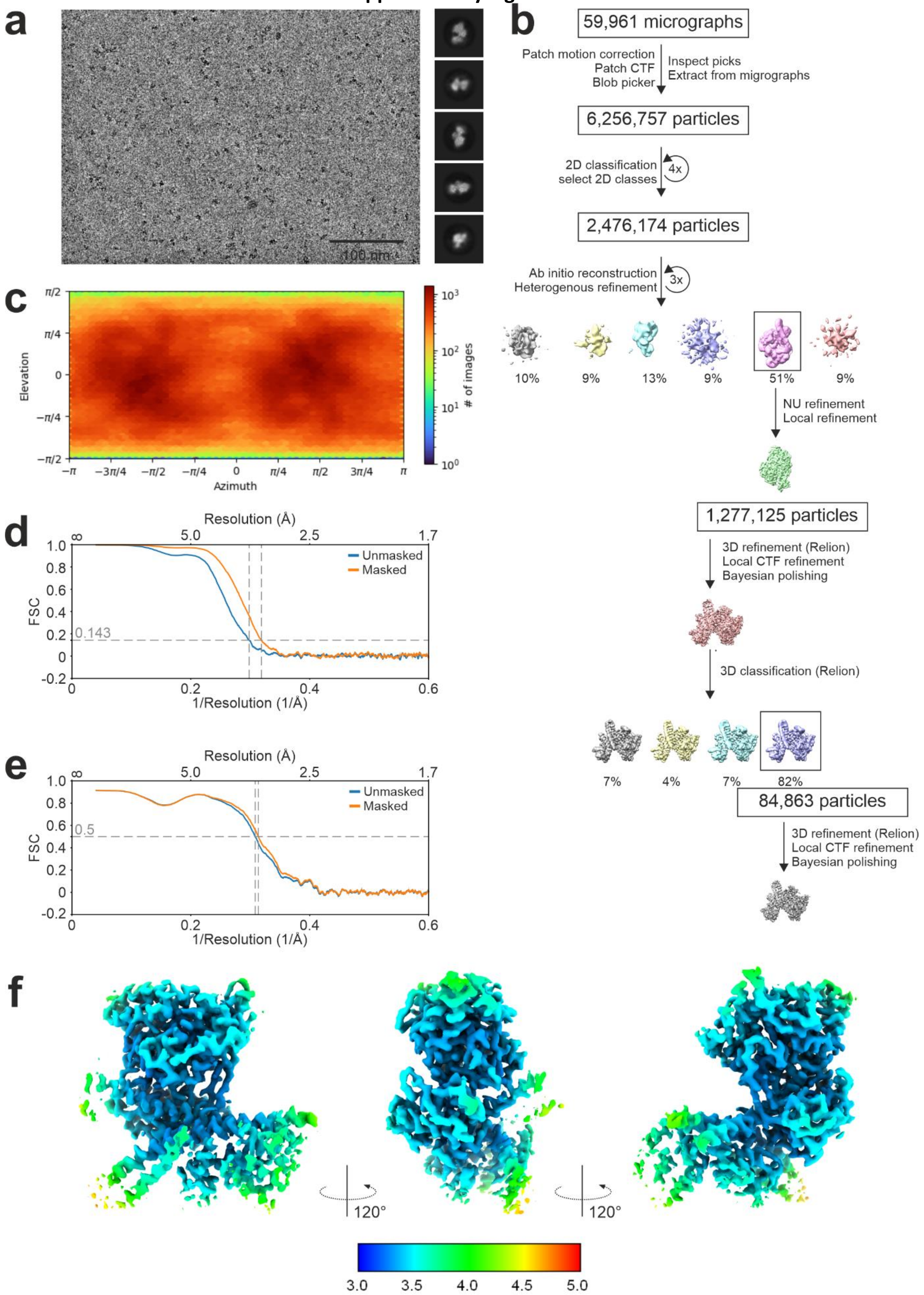

Supplementary Figure 2. Single particle Cryo-EM processing and reconstruction of human MICAL1.

(a) Representative micrograph and 2D classes of human MICAL1 particles.

(b) Flowchart outlining the cryo-EM data processing pipeline.

(c) Angular distribution heat map showing the particle projections used in the reconstruction.

(d) Fourier Shell Correlation (FSC) curve for the human MICAL1 reconstructions, comparing half-maps. The dashed line indicates the overall nominal resolution at the 0.143 FSC threshold, as calculated by Phenix.

(e) FSC curve comparing the model and map for the human MICAL1 reconstruction. The dashed line indicates the overall nominal resolution at the 0.5 FSC threshold, as calculated by Phenix.

(f) Local resolution map of the human MICAL1 reconstruction, with resolution color-coded as follows: blue (3.0 Å), cyan (3.5 Å), green (4.0 Å), yellow (4.5 Å), and orange (5.0 Å).

Supplementary Figure 3 – part1

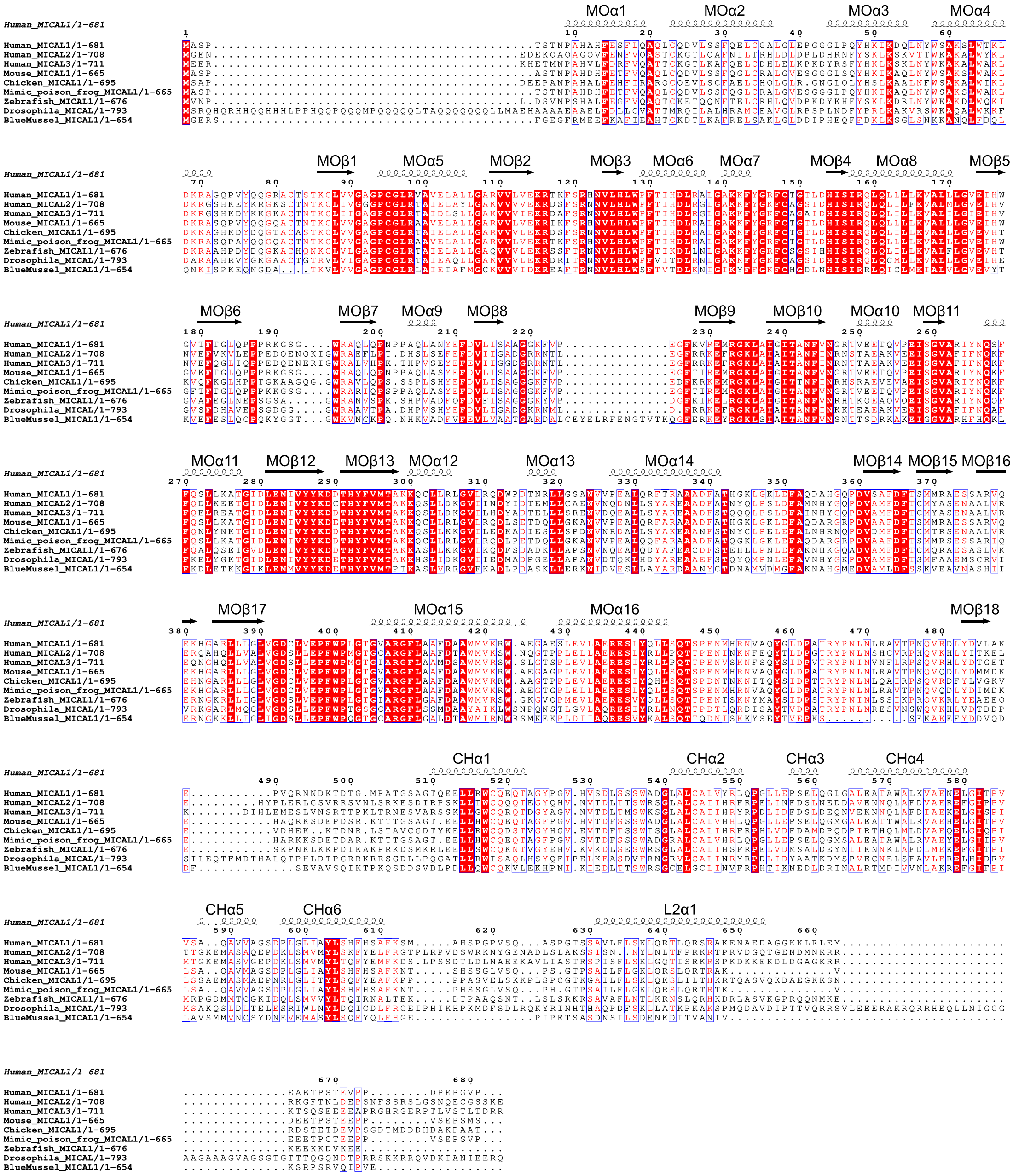

Supplementary Figure 3. Sequence alignment of diverse MICAL proteins

Structure-based sequence alignment of MICAL proteins from various species, focusing on domains critical for their function, including MO, CH, LIM, L2α1, and CC. For clarity, only sequences covering these domains are included, with the longer linker regions omitted except for the L2α1 helix. The alignment was constructed using KAlign[52] and has been formatted using ESPript[53].

Figure S3 – part2

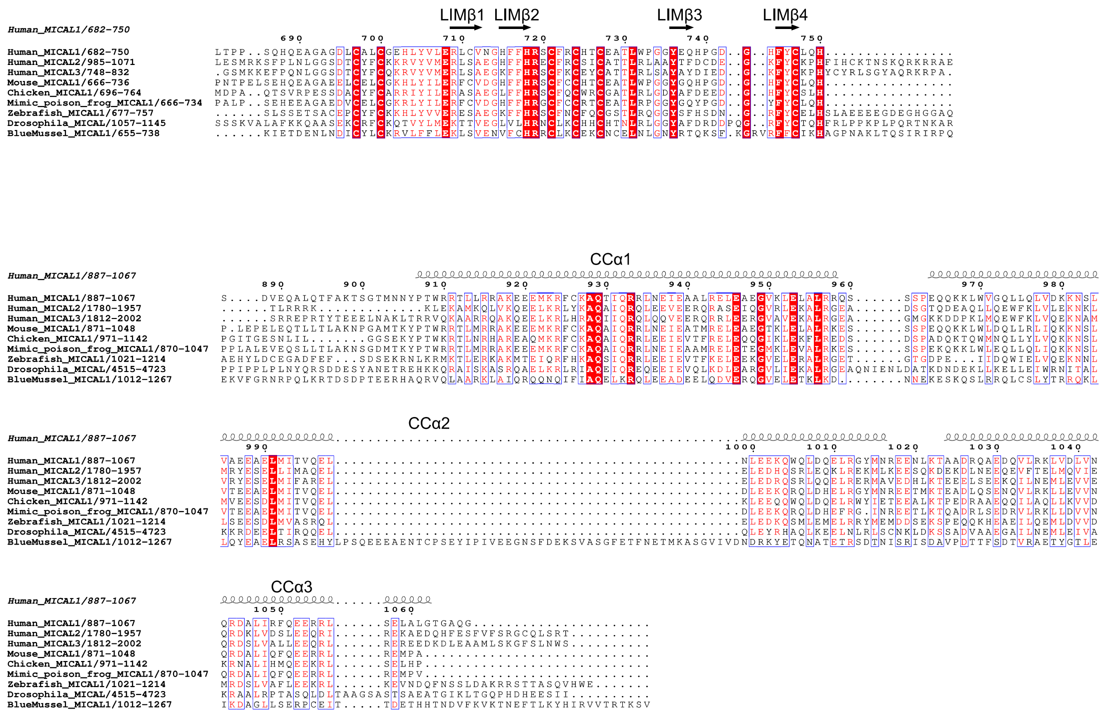

Supplementary Figure 3. Sequence alignment of diverse MICAL proteins

Structure-based sequence alignment of MICAL proteins from various species, focusing on domains critical for their function, including MO, CH, LIM, L2α1, and CC. For clarity, only sequences covering these domains are included, with the longer linker regions omitted except for the L2α1 helix. The alignment was constructed using KAlign[52] and has been formatted using ESPript[53].

Supplementary Figure 4

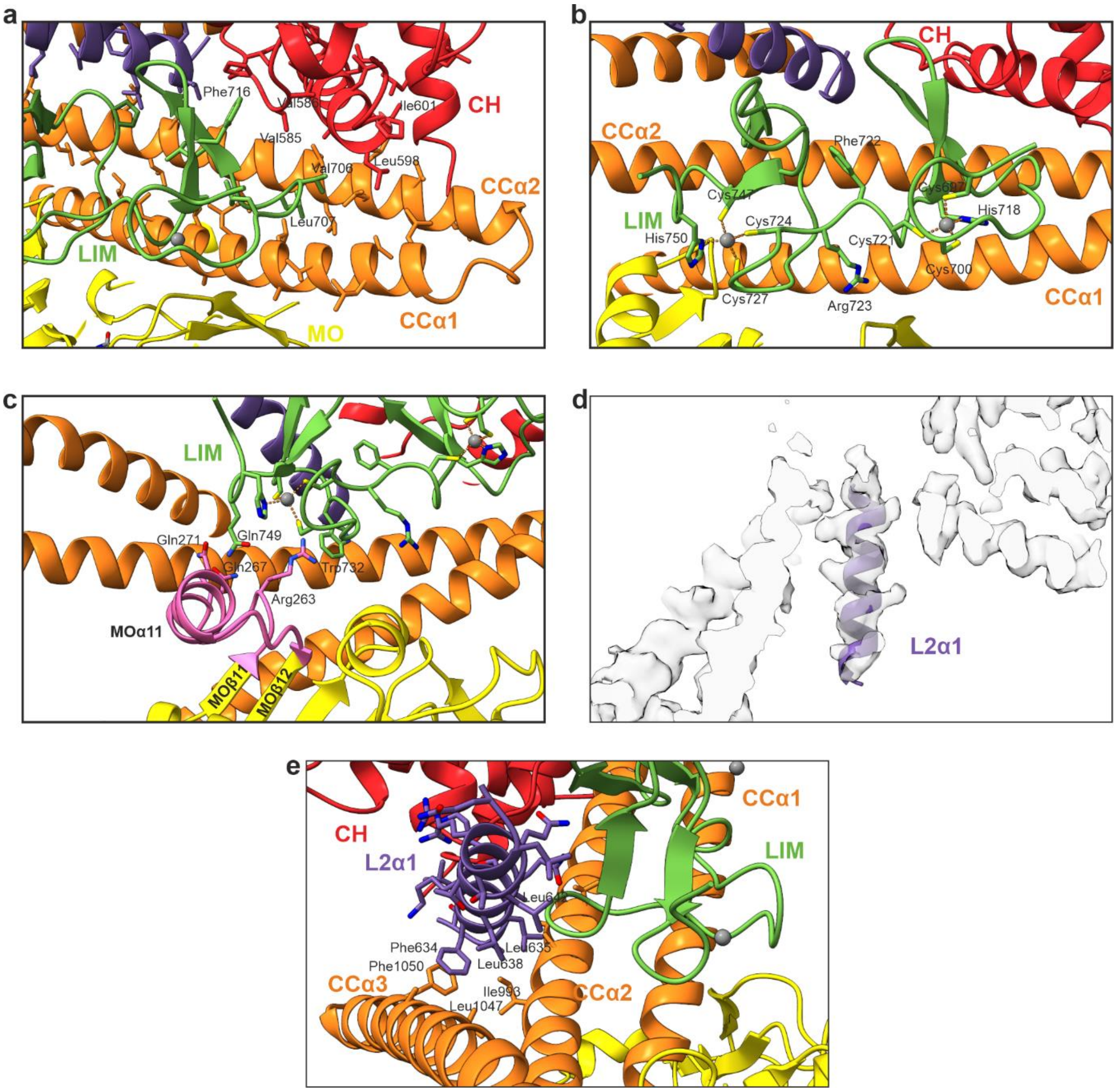

Supplementary Figure 4. Interactions between domains in human MICAL1

(a) The distal part of CCα1 and the proximal part of CCα2 are tightly packed through hydrophobic interactions predominantly involving isoleucine, leucine, and valine residues. This region, being particularly hydrophobic, serves as the binding site for the CH domain, which interacts with this area through its residues Val585, Leu598, and Ile601. The LIM domain binds centrally to both CCα1 and CCα2, also through hydrophobic contacts, primarily mediated by Val706 and Leu707. Additionally, hydrophobic residues such as Val585, Val586, Val706, and Phe716 are involved in the interactions between the LIM and CH domains.

(b) Close-up view of the LIM domain structure. The LIM domain consists of two contiguous Cys3His zinc finger motifs, separated by a Phe-Arg pair.

(c) Close-up view of the interaction between the LIM domain and the MO domain. The MO region involved in this interaction, highlighted in pink, is situated between MOβ11 and MOβ12, and includes the MOα11 segment.

(d) Close-up view of the cryo-EM map around the CH-L2α1-LIM-CC assembly.

(e) Close-up view of the hydrophobic interaction between the L2α1 helix and the central regions of CCα2 and CCα3.

Supplementary Figure 5

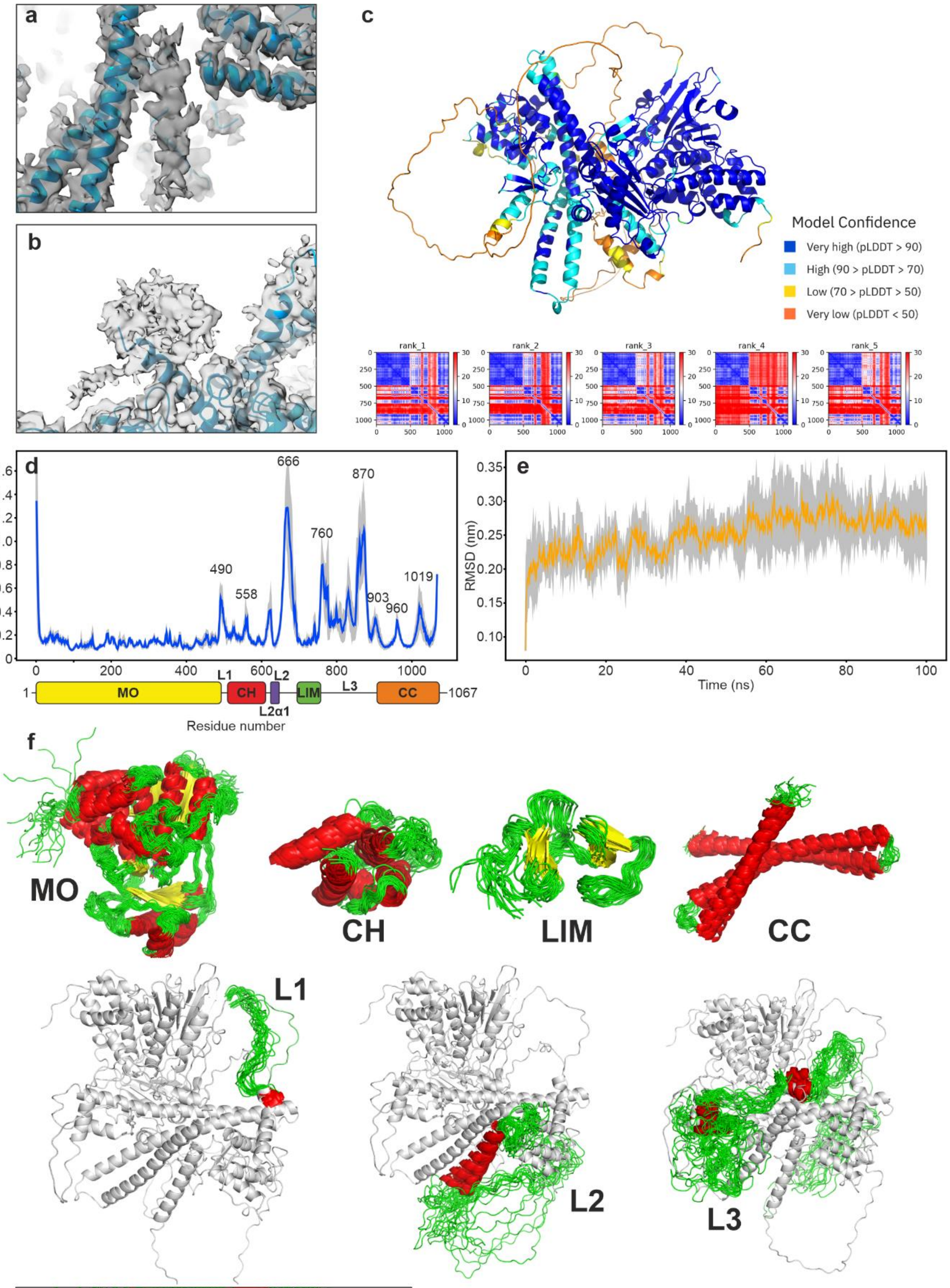

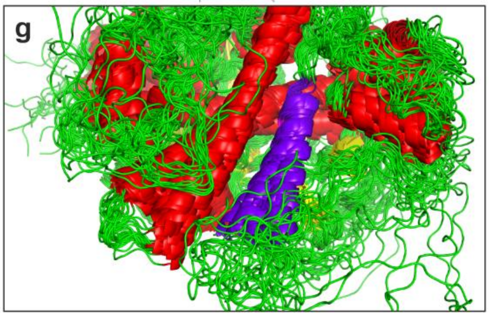

h

Source	Interaction	Target	Occupancy
ala-631	VDW	phe-1050	31,1%
val-632	VDW	ala-989	41,0%
phe-634	PIPISTACK	phe-1050	95,6%
phe-634	VDW	leu-1047	44,3%
leu-635	VDW	pro-733	43,6%
leu-635	VDW	ala-989	38,1%
leu-635	VDW	ile-993	37,7%
leu-635	VDW	met-992	50,9%
leu-638	VDW	gln-996	33,0%
gln-639	HBOND	gly-734	75,8%
gln-639	HBOND	gly-735	37,0%
gln-639	HBOND	tyr-736	47,6%
arg-646	VDW	leu-748	34,4%
glu-649	IONIC	lys-1036	54,9%

Supplementary Figure 5. Molecular dynamics simulation analysis of human MICAL1

(a) Cryo-EM density map corresponding to the L2α1 helix within the L2 linker region.

(b) Additional Cryo-EM density observed near the proximal part of CCα1. This bulk region likely represents an arrangement of small helices, with a narrow extension suggesting the presence of a peptide chain. While AlphaFold predicted this region to be proline-rich, the local low resolution prevented unambiguous model building or experimental confirmation of this segment.

(c) Ribbon representation of AlphaFold prediction of the linker regions in human MICAL1, supplemented with predicted local distance difference test (pLDDT) scores and predicted alignment errors (PAE).

(d) Average root mean square fluctuations (RMSF) of Cα atoms (blue) from three independent simulations, with standard deviation depicted in grey shading.

(e) Root mean square deviation (RMSD) of Cα atoms of the simulated protein structure compared to the initial cryo-EM structure. The average RMSD across the three runs is shown in orange, with the standard deviation depicted in grey. The average RMSD reaches a plateau, indicating system equilibration.

(f) Movement patterns of the four domains (MO, CH, LIM, and CC) and the linker regions throughout the 100 ns molecular dynamics simulation.

(g) Movement patterns of the L2α1 helix.

(h) Interactions of the L2α1 helix with the CH, LIM, and CC domains, along with their calculated occupancy throughout the molecular dynamics simulation.

Supplementary Figure 6

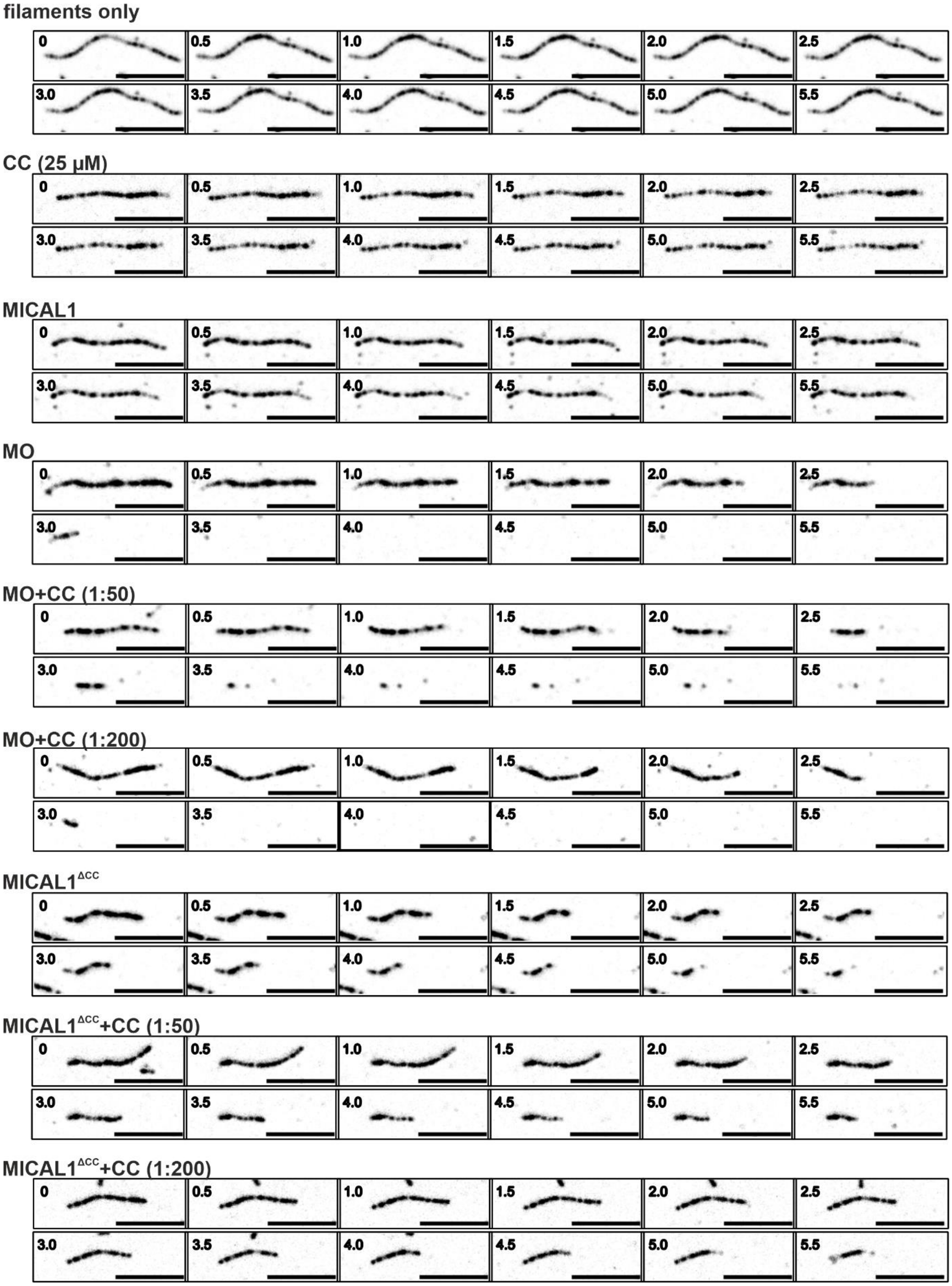

Supplementary Figure 6. Time-lapse TIRF microscopy images of a representative single actin filament, illustrating the impact of the purified CC domain on the depolymerization activity of MICAL1 variants. Scale bar, 5 µm, time is shown in minutes.

Supplementary Figure 7

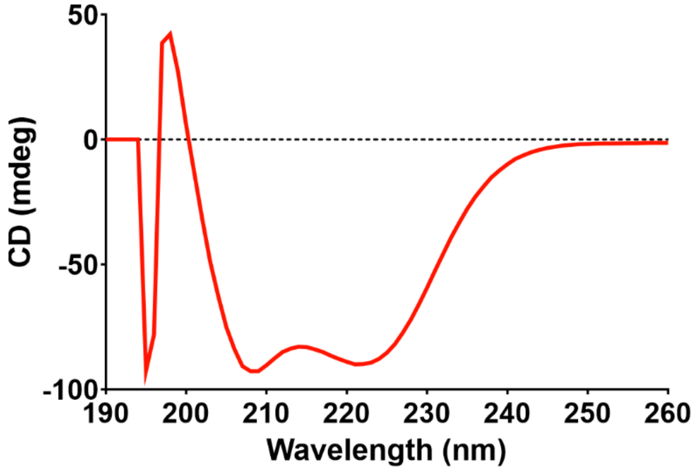

Supplementary Figure 7. CD spectra showing the helical arrangement of the CC domain.

Supplementary Figure 8 – part 1

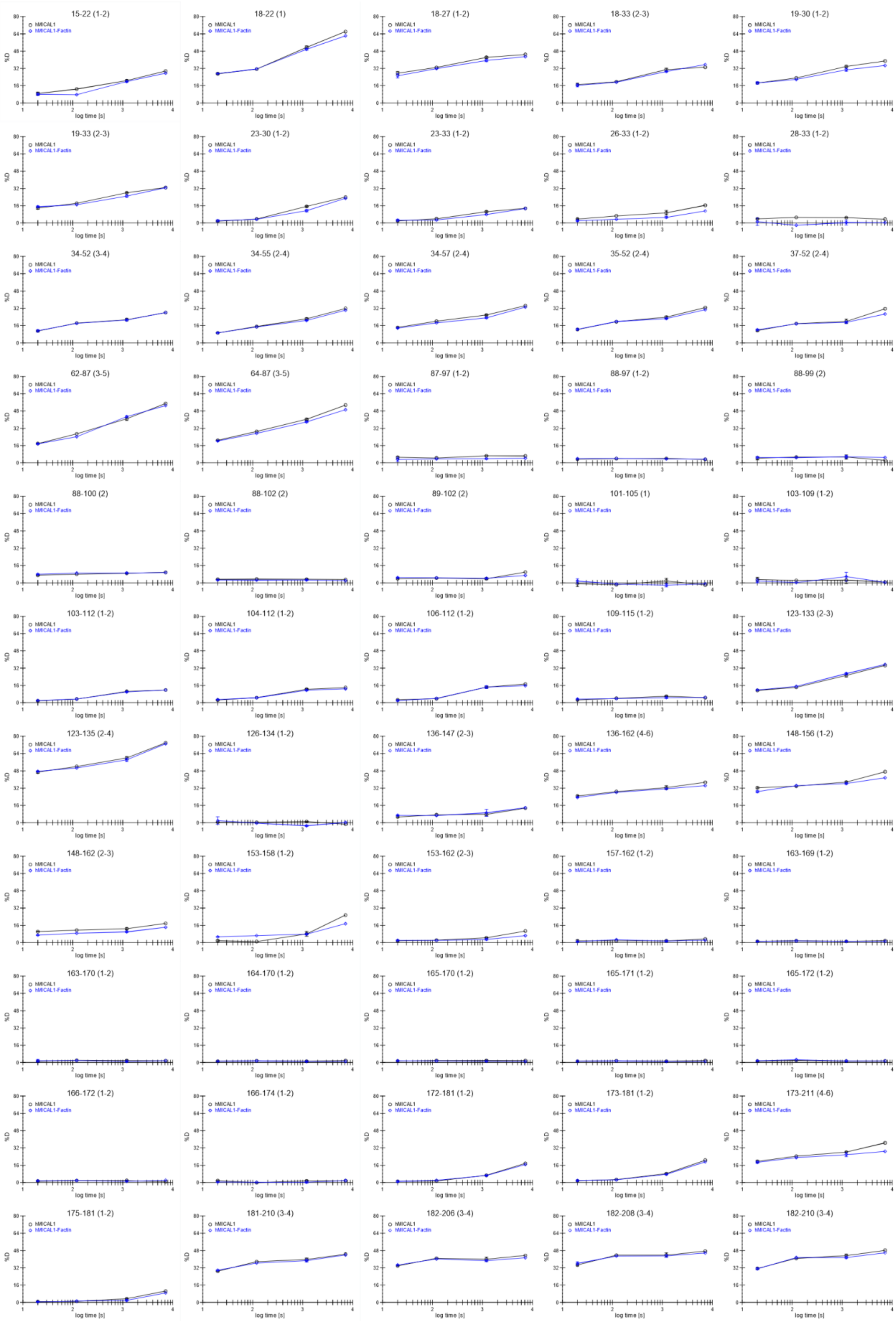

Supplementary Figure 8 – part 2

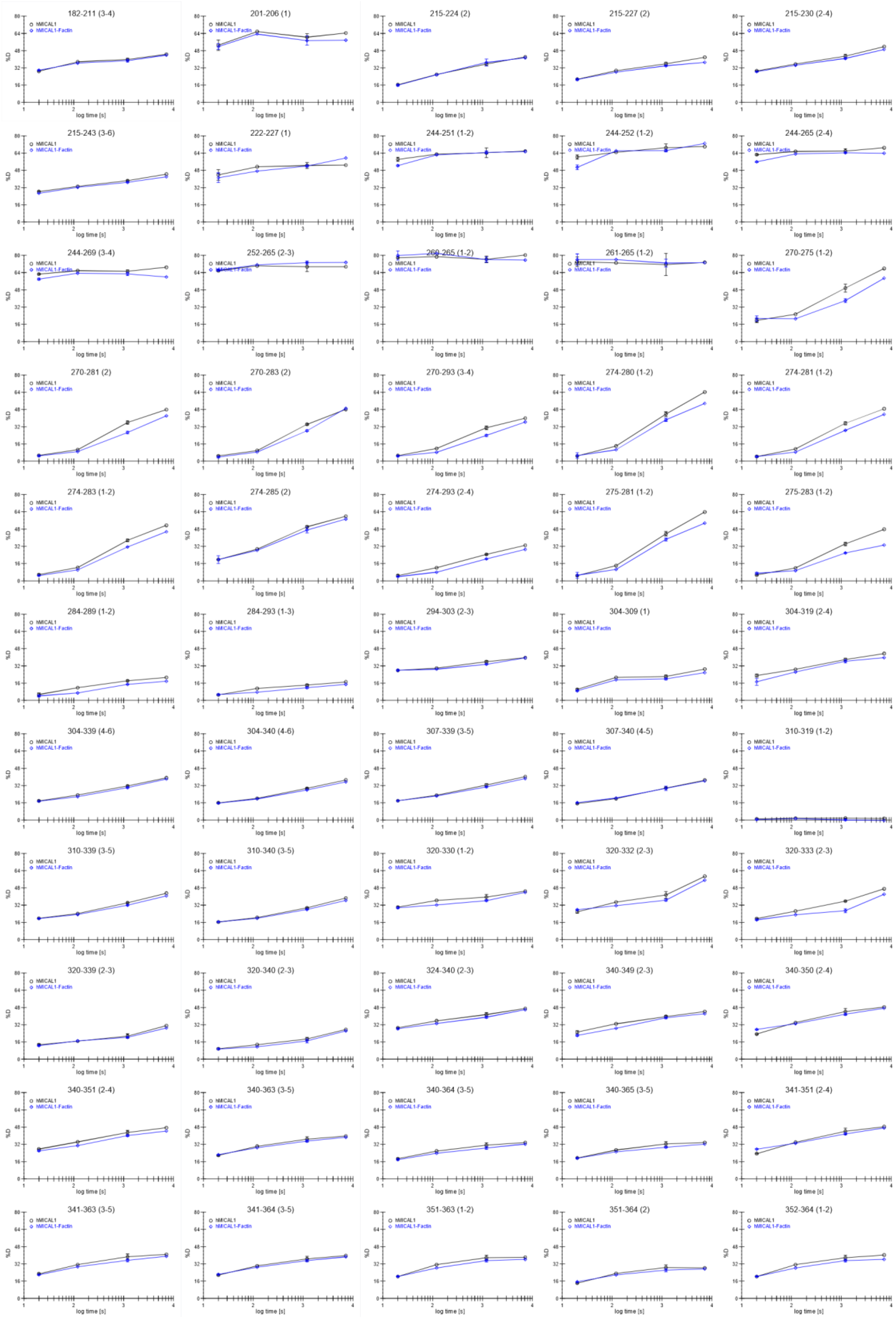

Supplementary Figure 8 – part 3

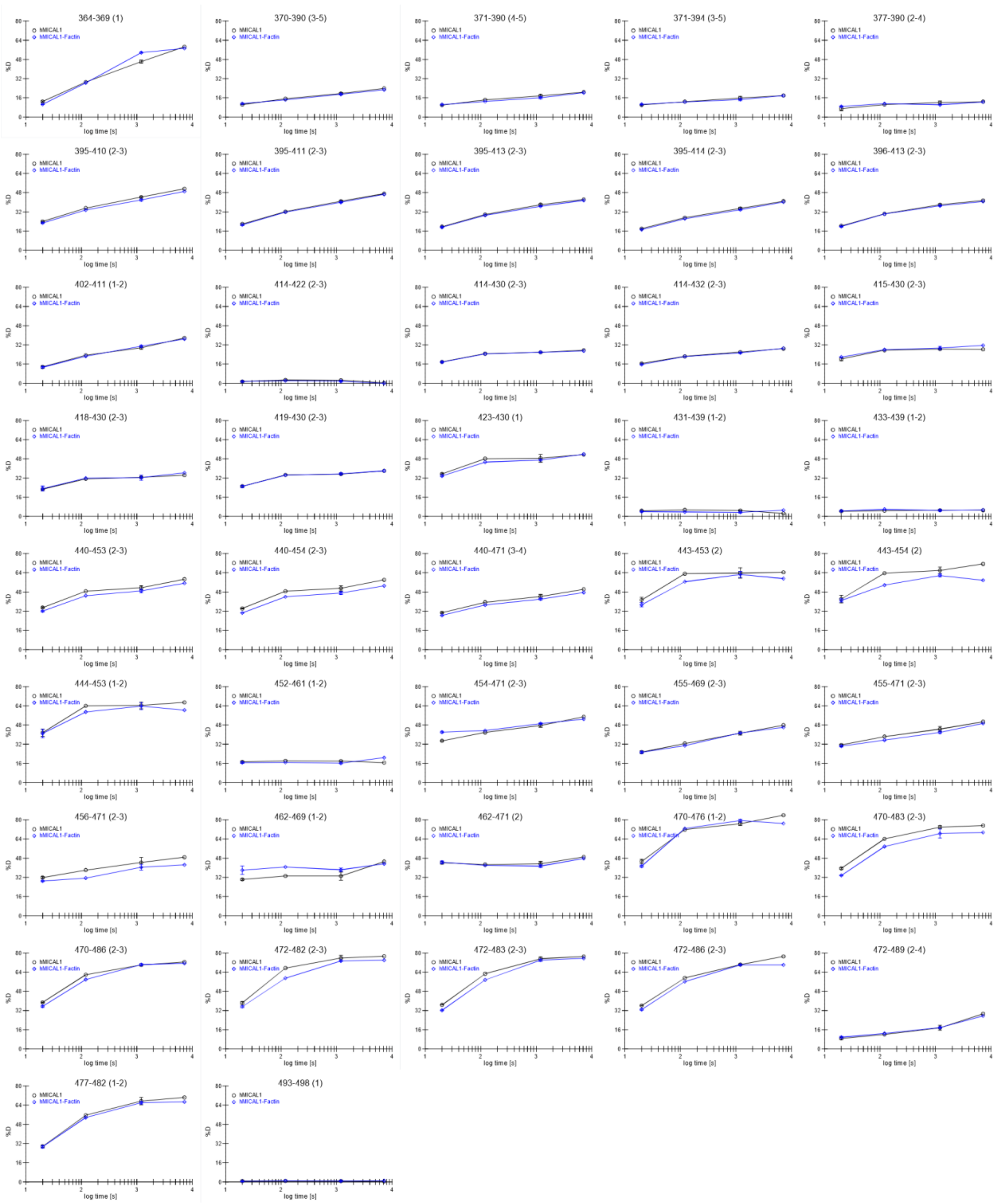

Supplementary Figure 8. Time-dependent deuterium uptake profiles for individual peptides of the MO domain, analyzed in the presence (blue) and absence of F-actin (black). The plots display the percentage of deuteration over time. Data are presented as the mean ± S.D. (n = 2).

Supplementary Table 1. Cryo-EM data collection, refinement and validation statistics

**Data Collection**	
Microscope	Titan Krios G1
Voltage (kV)	300
Detector	Bioquantum K3
Recording mode	Counting mode
Magnification	105,000
Movie/micrograph pixel size (Å)	0.834
Dose rate (e / Å^2/sec)	21
Number of frames per movie	40
Movie Exposure time (s)	2.5-3.0
Total dose (e/Å^2)	84
Defocus range (µm)	-1.4 to -2.8
Volta Phase Plate	no
**EM Data Processing**	
Number of movies/micrographs	59,961
Box size (px)	256
Particle number (total)	6,256,757
Particle number (post 2D)	2476174
Particle number (post 3D)	1,277,125
Particle number (for final map)	84,863
Symmetry	C1
dFSC (half maps; 0.143)	3.1
D99 (full/half1/half2)	2.6/1.7/1.7
Map sharpening B-factor (Å2)	-58.3
**Model Building and Validation**	
Initial model used	2BRY, 4TXI, 5LE0
**Model Composition**	
Non-hydrogen protein atoms	6498
Protein residues	815
**Ligands**	
Zn	2
FAD	1
**Bonds (RMSD)**	
Length (Å)	0.003
Angles (°)	0.477
**Validation score**	
MolProbity score	1.20
Clash score	4.16
Rotamer outliers (%)	0
d FSC model (0/0.143/0.5)	2.4/2.8/3.2
CC model-vs-map (masked)	0.87
**Ramachandran plot**	
Favoured (%)	98.01
Allowed (%)	1.86
Outliers (%)	0.12

Supplementary Table 2. HDX Summary

**Data Set**	**MICAL1-MO**	**MICAL1-MO / F-Actin complex**
HDX reaction details	20 mM HEPES-NaOH, pH_{read} 7.1/pD 7.5, 50 mM NaCl, 2 mM DTT, 20 °C	20 mM HEPES-NaOH, pH_{read} 7.1/pD 7.5, 50 mM NaCl, 2 mM DTT, 20 °C
HDX time course (min)	0.33, 2, 20, 120	0.33, 2, 20, 120
HDX control samples	Maximally-labeled control (MICAL1 MO)	Maximally-labeled control (MICAL1 MO)
Back-exchange (mean / IQR)	36.51% / 12.73%	
# of Peptides	152	
Sequence coverage	92%	
Average peptide length / Redundancy	14.5 / 4.7	
Replicates (biological or technical)	3 - biological (for 0.33, 20 min and maximally-labeled ctrl)	3 - biological (for 0.33, 20 min and maximally-labeled ctrl)
Repeatability	1.07 %D (average standard deviation)	0.89 %D (average standard deviation)
Significant differences in HDX (delta HDX > X D)	1.96 %D	

Supplementary Table 3. List of oligonucleotides

CONSTRUCT NAME	FORWARD PRIMER	REVERSE PRIMER
MICAL1 (RESIDUES M1-G1067)	ATTCTAGAATGGCTTCACCTACCTCCACC	ATTTAATTAATGGTGGTGATGGTGATGGTGGTGGCTACCGGGGCCCTGGAACAGCACCTCCAGGGTGCCCTGGGCCCCTGTCCCCAAGGC
MICAL1$^{\Delta MO}$ (RESIDUES A508-G1067)	TATTCTAGAGCCACCATGGCAGGCACCCAGGAGGAG	ACGCACAGAATCTAGCGCTT
MICAL1$^{\Delta CC}$ (RESIDUES M1-A917)	ATTCTAGAATGGCTTCACCTACCTCCACC	AATTAATTAATGGTGGTGATGGTGATGGTGGTGGCTACCGGGGCCCTGGAACAGCACCTCCAGGCTGCCTTCCTTTTTGTGGTC
MICAL1 MO (RESIDUES T7-E489)	TCTAGAAAGGAGAATATGGCTTCACCTACCTCCACC	ATTTAATTAATGGTGGTGATGGTGATGGTGGTGGCTACCGGGGCCCTGGAACAGCACCTCCAGGGTCTCCTTGGCTAGCACATCATACAG
MICAL1 CC (RESIDUES K918-G1067)	ATTACCGGTAAGGAGGAGGAGATGAAGAGGT	ATAGGTTAATTAATGCCCTGGGCCC
MICAL1 CC AVI TAG (RESIDUES K918-G1067)	ATTACCGGTAAGGAGGAGGAGATGAAGAGGT	Reverse 1: GAAGATGTCGTTCAGGCCGCTTCCTCCGCTTCCTCCGCCCTGGGCCCCTGTCCCC<br>Reverse 2: CCTTAATTAATTTCGTGCCATTCGATTTTCTGAGCCTCGAAGATGTCGTTCAGGCCGC
MICAL1 R933A	AAGGCCCAGACCATCCAAGCCCGACTAAATGAGATTGAG	CTCAATCTCATTTAGTCGGGCTTGGATGGTCTGGGCCTT

Supplementary Table 4. Summary of molecular dynamics simulation setup

Simulation box dimensions	15.3 nm
Total number of atoms	337 065
Total number of water molecules	6 618
NaCl concentration	150 mM
