# Supplementary material for: Structural basis of MICAL autoinhibition
Source: Nat Commun. 2024 Nov 12;15:9810. doi: 10.1038/s41467-024-54131-2 (PMC11557892; doi:10.1038/s41467-024-54131-2)
Supplement: Supplementary file 2 — Reporting Summary [file 41467_2024_54131_MOESM2_ESM.pdf]

nature portfolio

Corresponding author(s): Daniel Rozbesky

Last updated by author(s): Oct 31, 2024

Reporting Summary

Nature Portfolio wishes to improve the reproducibility of the work that we publish. This form provides structure for consistency and transparency in reporting. For further information on Nature Portfolio policies, see our Editorial Policies and the Editorial Policy Checklist.

Statistics

For all statistical analyses, confirm that the following items are present in the figure legend, table legend, main text, or Methods section.

n/a	Confirmed	
☐	☒	The exact sample size (n) for each experimental group/condition, given as a discrete number and unit of measurement
☐	☒	A statement on whether measurements were taken from distinct samples or whether the same sample was measured repeatedly
☐	☒	The statistical test(s) used AND whether they are one- or two-sided<br>*Only common tests should be described solely by name; describe more complex techniques in the Methods section.*
☒	☐	A description of all covariates tested
☒	☐	A description of any assumptions or corrections, such as tests of normality and adjustment for multiple comparisons
☐	☒	A full description of the statistical parameters including central tendency (e.g. means) or other basic estimates (e.g. regression coefficient) AND variation (e.g. standard deviation) or associated estimates of uncertainty (e.g. confidence intervals)
☐	☒	For null hypothesis testing, the test statistic (e.g. F, t, r) with confidence intervals, effect sizes, degrees of freedom and P value noted<br>*Give P values as exact values whenever suitable.*
☒	☐	For Bayesian analysis, information on the choice of priors and Markov chain Monte Carlo settings
☒	☐	For hierarchical and complex designs, identification of the appropriate level for tests and full reporting of outcomes
☒	☐	Estimates of effect sizes (e.g. Cohen's d, Pearson's r), indicating how they were calculated

Our web collection on statistics for biologists contains articles on many of the points above.

Software and code

Policy information about availability of computer code

Data collection	SerialEM, Octet BLI Discovery 12.2, GROMACS V2020.3 with the CHARMM36m, ASTRA Software (Wyatt Technology - v8.0.3),
Data analysis	CryoSparc v3.1.1, Relion 3.0, Octet Analysis Studio 12.2.0.20, Prism 10.0.2 (GraphPad), ImageJ2 v2.14.0, GROMACS v2020.3 with the CHARMM36m, ASTRA Software v8.0.3 (Wyatt Technology), Bio-Rad Image Lab Software v6.1, PYMOL (Schrodinger, LLC, v1.8.6.2), ChimeraX 1.4, ESPRIPT 3.0, PDBePISA, PDBeFold, COOT (v.0.8.9.1). KAlign v3.0, Consurf, DataAnalysis (v. 5.3, Bruker Daltonics), DeutEx software v1.2, MSTools v1.4, PyMOL 2.5.5, MASCOT (v. 2.7, Matrix Science), Chronos software v3.2 (AxelSemrau), WinHydroPRO v3.0

For manuscripts utilizing custom algorithms or software that are central to the research but not yet described in published literature, software must be made available to editors and reviewers. We strongly encourage code deposition in a community repository (e.g. GitHub). See the Nature Portfolio guidelines for submitting code & software for further information.

Data

Policy information about availability of data

All manuscripts must include a data availability statement. This statement should provide the following information, where applicable:

- Accession codes, unique identifiers, or web links for publicly available datasets
- A description of any restrictions on data availability
- For clinical datasets or third party data, please ensure that the statement adheres to our policy

The cryo-EM map has been deposited in the Electron Microscopy Data Bank (EMDB) under accession code EMD-50026 [https://www.ebi.ac.uk/emdb/EMD-50026] and the corresponding model coordinates have been deposited in the Protein Data Bank (PDB) under accession number 9EWY [https://doi.org/10.2210/pdb9EWY/

pdb]. For molecular dynamics simulation, the initial coordinates, simulation input files and the final coordinate output file generated in this study have been deposited in the Zenodo public repository under accession code 13987364 [https://doi.org/10.5281/zenodo.13987364]. The HDX data have been deposited to the ProteomeXchange repository with the dataset identifier PXD057312 [http://www.ebi.ac.uk/pride/archive/projects/PXD057312].

Research involving human participants, their data, or biological material

Policy information about studies with human participants or human data. See also policy information about sex, gender (identity/presentation), and sexual orientation and race, ethnicity and racism.

Reporting on sex and gender	N/A
Reporting on race, ethnicity, or other socially relevant groupings	N/A
Population characteristics	N/A
Recruitment	N/A
Ethics oversight	N/A

Note that full information on the approval of the study protocol must also be provided in the manuscript.

Field-specific reporting

Please select the one below that is the best fit for your research. If you are not sure, read the appropriate sections before making your selection.

[x] Life sciences [ ] Behavioural & social sciences [ ] Ecological, evolutionary & environmental sciences

For a reference copy of the document with all sections, see nature.com/documents/nr-reporting-summary-flat.pdf

Life sciences study design

All studies must disclose on these points even when the disclosure is negative.

Sample size	No statistical methods were used to predetermine sample size in this study. For in vitro biochemical assays, sample sizes were chosen based on common experimental practice, and all experiments were performed with at least two-three independent repeats to ensure reproducibility and reliability of the results. The chosen sample sizes were deemed sufficient for detecting the expected biochemical effects and for the objectives of the study.
Data exclusions	Data points were excluded when there was a technical mistake during the procedure of the experiment.
Replication	All BLI binding experiments were performed in independent duplicates, including the preparation of dilutions. All MD simulations were conducted in triplicate. The F-actin depolymerization assay was performed in at least three independent replicates. Single actin filament TIRF microscopy was performed in at least three independent replicates. The HDX experiment was prepared in triplicate. All attempts at replication were successful.
Randomization	For cryo-EM data processing, the datasets were randomly divided into two halves (halfmaps) to determine the nominal resolution using Fourier Shell Correlation (FSC). Randomization was not required or applicable for the other assays in this study, as they were designed to focus on specific, controlled experimental conditions that did not necessitate random assignment.
Blinding	Blinding was not relevant to our study.

Reporting for specific materials, systems and methods

We require information from authors about some types of materials, experimental systems and methods used in many studies. Here, indicate whether each material, system or method listed is relevant to your study. If you are not sure if a list item applies to your research, read the appropriate section before selecting a response.

Materials & experimental systems

n/a	Involved in the study
☐	☒ Antibodies
☐	☒ Eukaryotic cell lines
☒	☐ Palaeontology and archaeology
☒	☐ Animals and other organisms
☒	☐ Clinical data
☒	☐ Dual use research of concern
☒	☐ Plants

Methods

n/a	Involved in the study
☒	☐ ChIP-seq
☒	☐ Flow cytometry
☒	☐ MRI-based neuroimaging

Antibodies

Antibodies used	anti-biotin antibody (#B3640, Merck) - Polyclonal primary antibody anti biotin produced in goat
Validation	We used anti-biotin antibody to immobilize biotinylated actin filaments to the glass surface. The antibody has been validated for use by the manufacturer, as indicated in the product datasheet. We relied on the validation information provided by the manufacturer. No additional in-house validation was performed for this antibody.

Eukaryotic cell lines

Policy information about cell lines and Sex and Gender in Research

Cell line source(s)	Sf9 cells (Spodoptera frugiperda) from ATCC CRL-1711
Authentication	None of these cell lines were explicitly authenticated.
Mycoplasma contamination	All cell lines tested negative for mycoplasma.
Commonly misidentified lines (See ICLAC register)	No ILAC listed cell lines that are commonly misidentified were used in this study.

Plants

Seed stocks	N/A
Novel plant genotypes	N/A
Authentication	N/A
